# Something “New under the Sun!” Negro Testimony vs. the Science of Medicine, &c.

**Published:** 1857-09

**Authors:** J. Boring

**Affiliations:** Professor of Obstetrics in the Atlanta Medical College


### ARTICLE II.

*Something "new under the Sun!" Negro Testimony* vs. *the Science of Medicine! A learned Professor of Medicine introducing an old African Slave to his Medical Class for the purpose of eliciting his Experience and Opinion in the settlement of a controverted physiological question.* By J. BORING, M. D., Professor of Obstetrics in the Atlanta Medical College.

My attention has recently been called to an editorial notice in the *Nashville Journal of Medicine and Surgery*, for August, 1857, of the Lecture delivered by me, on the subject of the "Use and Abuse of Tobacco," at the opening of the present course of Lectures in the "Atlanta Medical College," in which, while the writer shrinks from being understood as "defending the use of Tobacco," or as being the apologist of those who do use it," he *disingenuously seeks* to cast opprobrium upon the Lecture, and parry its force, by reference to a former discussion, in which he *had* taken the very position here disclaimed, and in the defence of which he had introduced to his Medical Class an old African slave, for the purpose of invoking his experience and *opinion.*

He tells us, that having introduced "Old George," who was then "ninety-seven years of age, he asked him, 'What a man ought to do, and live on, to attain his great age, and yet be hale and strong." Interesting teacher and pupil!—The old teacher answered, "he ought to eat plenty of bacon, corn bread, and cabbage, and work from Monday morning until Saturday night in the open field." The student then asked his sable teacher,—

"Do you drink any sort of spirits?"

*George*—"Not much—never did drink much; never drunk."

"Do you use tobacco?"

*George*—"Yes Sir."

"How do you use it?"

*George*—"Smoking it in pipe and chewing it."

"Do you use much?"

*George*—"Always smoking or chewing, except when eating."

"What! not when you are sleeping?"

*George*—"No! I don't *chew* it while I sleep, but it is *there* in my mouth, ready for me when I wake. I always go to sleep with tobacco in my mouth."

"How long have you used tobacco?"

*George*—"About *ninety years.*"

"You have a wife?"

*George*—"Yes, I have my seventh wife now."

"How many children have you had?"

*George*—"I don't know how many I have buried, but a great many. I have only *thirty-seven* living."

Here the instructions concluded, the pupil, evidently being well instructed, and satisfied with his attainments. The impressions of this *African eloquence* seem, indeed, indelibly written, and singularly influential even to the present moment. But are we to understand that the chewing and smoking tobacco, the number of wives *enjoyed*, or the numerous children begotten, or the *whole*, together with bacon, corn-bread and cabbage, and hard work, furnish the means of attaining so great an age with vigor, such as George developed? I suppose the whole are to be taken into the account, especially the large number of wives, or *women*, to whom the *old man had* enjoyed access, and the remarkable proficiency developed of begetting children, since they are so particularly enumerated, and some of them italicised by the (then) student, now *learned*

*professor*, and that he who would share like blessings with "good old George," must adopt *his means* for their "attainment." Is the proposition adopted? Having been denied the advantages of *such* instructions, I cannot venture on the one hand to adopt the doctrines; nor, on the other, an attempt to answer the *arguments* by which they are so forcibly inculcated. I surrender the point without discussion. It is absolutely inapproachable.

This, however, is by no means the first instance in which I have been *routed* by unanswerable arguments of *the kind.*

A short time since, while engaged in a case of midwifery, in the country, the placenta being extracted, a company of wise-looking old matrons demanded that it should be burned, affirming from "experience," that unless this was done the patient would "flood." The *argument* was resistless, I *surrendered*, and forthwith pitched the after-birth into the fire.—While it was broiling and hissing on the coals, one of the company, an *expert* in the "third degree" of *self-importance*, said to me, "Doctor, do you know *jist* what will stop flooden?" I was "stumped," and asked her if *she* knew of such a remedy. "Well," she said, "let the woman swallow *jist two draps* of the blood in that ar cord, and thar's an eend o' flooden." It was too much. I was vanquished, and yielded the field.

